# Female Genital Arousal: A Focus on *How* Rather than *Why*

**DOI:** 10.1007/s10508-020-01792-x

**Published:** 2020-07-15

**Authors:** Marieke Dewitte

**Affiliations:** Department of Clinical Psychological Science, Maastricht University, Universiteitssingel, 40, 6229 ER Maastricht, The Netherlands

## Introduction

Sexual arousal is commonly conceived as a dynamic, multi-component process that is experienced and expressed through different cognitive, motivational, and physiological response systems (Janssen, 2011; Janssen, Everaerd, Spiering, & Janssen, 2000). Yet, the Target Article by Lalumière, Sawatsky, Dawson, and Suschinsky (2020) features only one specific component of sexual arousal, namely the physiological response. Inspired by evolutionary theory, the preparation hypothesis has been forwarded to explain a common finding in sex research: that genital arousal in women can be automatically activated in response to a variety of sexual stimuli that are non-specific in terms of sexual attraction (Suschinsky & Lalumière, 2011). Even stimuli that are unappealing, undesired, aversive, and nonconsensual have been found to trigger a genital arousal response in women (Chivers, 2005; Chivers, Rieger, Latty, & Bailey, 2004; Laan & Everaerd, 1995; Suschinsky & Lalumière, 2011). As these stimuli do not necessarily induce sexual excitement, it appears that genital arousal can be paired with negative affect and low subjective sexual arousal (Chivers, Seto, Lalumière, Laan, & Grimbos, 2010). According to the preparation hypothesis, women have developed a reflexive lubrication response to any sexual cue that could lead to penetration in order to prepare and hence protect the vagina from potential injury (Suschinsky & Lalumière, 2011). Genital arousal would thus primarily serve a protective (i.e., avoidance-driven) function and unfold independently from subjective sexual arousal, which is more likely driven by a pleasure (i.e., approach-oriented) motivational focus.

The preparation hypothesis is unclear about the (evolutionary adaptive) function of subjective sexual arousal and how this relates to genital arousal. It seems that the hypothesis is more strongly directed toward explaining low cue-specificity of genital vasocongestion, rather than explaining the weak correlation between different components of sexual arousal. In the Target Article, several references are made to sexual interest, but subjective sexual arousal needs to be differentiated from sexual desire or interest. The latter includes a motivational component, namely fueling the willingness to act on physical and subjective signs of sexual arousal (Both, Everaerd, & Laan, 2007; Toates, 2009). Subjective sexual arousal, on the other hand, refers to an integrative process of becoming aware of physical signs of arousal, attentional processes, and cognitive elaboration (Both et al., 2007; Janssen et al., 2000). This cascade of physical, cognitive, and motivational processes would operate in the same way for men and women, which is not well captured by evolutionary hypotheses that are limited to explaining only fragments of sexual behavior, most often in a static, hard-wired, binary, and sex-differentiated way.

The central question is: Do we actually need an evolutionary hypothesis to explain the function, underlying dynamics, and gender differences in genital arousal responding? Evolutionary theory has been criticized from many different angles, raising concerns about testability and falsifiability, overly broad assumptions, stability and rigidity rather than flexibility, ignoring the role of learning processes and contextual influences, disregarding complexity and individual differences, as well as lack of practical implications (Confer et al., 2010; Hankin, 2013; Smith & Konik, 2011). The preparation hypothesis is not immune to some of this criticism and may therefore lack unique explanatory power. Maybe the question of *why* women generate an automatic lubrication response to stimuli that are not necessarily liked or wanted is only relevant when combining it with the question *how* this may occur. To answer this question, we need other theoretical models that examine the proximal determinants of genital arousal, its relation to other components of sexual responding, and its practical implications in terms of sexual function and well-being. There are many other theories available that can generate more specific predictions about the activation and regulation of genital arousal rather than endorsing generic hypotheses that can account only for stereotypical response patterns that are insensitive to contextual influences. I will elaborate on some of these theories below.

## Sexual Arousal as a Multi-Determined, Multi-Component, and Multi-Level Process

A prominent idea in recent sex research is that sexual arousal does not arise spontaneously but is triggered by internal and external stimuli and involves a cascade of cognitive-motivational processes (Janssen, 2011; Janssen et al., 2000). According to incentive motivation and information processing accounts (Both et al., 2007; Janssen et al., 2000), sexual arousal is triggered by a stimulus that pre-attentively captures attention and is automatically appraised as sexually meaningful. Such automatic appraisal automatically evokes a genital arousal response, which then motivates people to maintain their attention to the sexual stimulus and cognitively elaborate (i.e., consciously appraise) on it. When this results in a positive evaluation (reinforced by memories of positive sexual events) and the expectation of reward, a subjective sense of arousal is experienced, which further increases genital and subjective arousal, resulting in a full-blown sexual response. These ongoing sexual responses may then trigger the desire and motivation to actually engage in sexual activities (see also Öhman, 1993; Toates, 2009).

Several important implications can be drawn from this model. First of all, genital arousal is not only involved in the initiation of sexual responding but also plays an important role in the regulation of the sexual response. In other words, lubrication not only functions to allow penetration and make it less painful, it actually becomes part of the stimulus event itself and feeds back into the sexual response (Janssen et al., 2000). Recent studies using an intravaginal balloon that simulates the vaginal pressure that women experience during penetration have shown that the genital sensations during pressure induction are appraised as more pleasant and less painful when experienced in a sexually arousing context (Dewitte, Schepers, & Melles, 2018; Melles, Dewitte, Ter Kuile, Bonnemayer, & Peters, 2018). This points toward the important role of context, subjective sexual arousal, and regulatory processes to prevent painful sexual responding. The regulatory function of genital arousal and the importance of subjective sexual arousal for painless sex are not well addressed by the preparation hypothesis.

A second important implication of this model is that the processing of a sexually competent stimulus may trigger emotion regulation strategies (Everaerd, 1988; Frijda & Mesquita, 1998). When framing sexual arousal as an emotion, we need to consider that emotions are inherently multi-level, multi-component processes that are experienced and expressed through changes in different response systems. Regulatory efforts can be directed at various parts of the emotion process, altering, magnifying, or inhibiting one or more components of the emotional response (Gross, 1998, 2002). Within the emotion literature, it is thus quite common to find discrepancies between different components of emotional responding (Gross, 1998). In other words, low correlations between physiological and subjective indices of sexual arousal can be understood in light of the dynamical and complex nature of sexual emotion regulation. This argues against a static prediction on the role of genital arousal responses to sexual cues.

Another important benefit of the multi-component model of sexual arousal is the idea that sexual responding depends on both automatic and controlled processes and that the different components of sexual arousal are under the control of different processes (Bush, 2001; Everaerd, Janssen, & Spiering, 2003). Drawing on dual process models, it has been argued that genital arousal is driven by reflexive and associative processes that are characterized by mere activation and can be triggered independently of values and beliefs. Subjective sexual arousal, on the other hand, would be determined by reflective and propositional processes that are based on truth values and validation of beliefs (Strack & Deutch, 2004). This does not, however, imply that genital arousal is a purely passive and reflexive process. It has been demonstrated that women can have some degree of voluntary control over their physiological sexual responses as measured in a laboratory setting (Prause, Barela, Roberts, & Graham 2013). Automatic and controlled processes can operate in parallel, synchronously, or in conflicting ways, which may result in discordance between sexual (arousal) responses (Chivers et al., 2010). Research has suggested that gender differences are more pronounced at controlled rather than automatic levels of sexual processing, which might explain why gender moderates the association between genital and subjective sexual arousal (Dewitte, 2016). Although the sexual system is assumed to function similarly in men and women, it is likely that differences exist in the type of stimuli that trigger the system, the ease with which the system gets activated, and the relative importance of certain regulatory processes in relation to different motivational triggers (Dewitte, 2015; Rupp & Wallen, 2007). Furthermore, when conceptualizing the sexual system as a dynamic emotion regulation device (Gross, 1998, 2002), it is plausible that gender differences do not appear on all process stages. Hence, decomposing sexual arousal into its component features, namely attention, appraisal, and motivation, and studying these at different levels of stimulus accessibility and responding (i.e., automatic versus controlled) allows a more detailed analysis of the process stages at which gender differences can be expected and what the source of these gender differences is.

## Sexual Arousal as a Dynamic and Malleable Process: The Role of Context

When assuming that the sexual system operates as an emotion regulation device, it is important to realize that emotion regulation is not a linear process (Freeman, 2000). Accordingly, research on gender differences in sexual arousal needs to consider the moderating impact of internal (e.g., motivational) and contextual influences (Dewitte, 2015; Rupp & Wallen, 2007; Timmers, Dawson, & Chivers, 2018) instead of treating these differences as stable and immutable entities (Conley, Moors, Matsick, Ziegler, & Valentine, 2011; Hyde, 2005). This implies that within-gender variation might be larger than between-gender variation, an idea that cannot be accounted for by evolutionary hypotheses (Petersen & Hyde, 2010). The problem with explaining women’s non-specific automatic genital arousal from an evolutionary perspective is its rigid focus on stereotypical gender differences, thereby yielding a status quo and not recognizing potential overlap. Moreover, the preparation hypothesis is less female-friendly than it seems because the primary function of female sexual responses is reduced to vaginal penetration, which entails the risk of using male sexuality as a benchmark, constraining the sexual responses of women by their biological function, and assuming that sexual pleasure is not necessarily adaptive as it has no evolutionary or protective function. In other words, genital arousal is defined as an avoidance-oriented response, driven by the need to prevent a negative outcome rather than pursuing a positive endpoint, which endorses a rather patriarchal view on women as passive victims of nature.

Another important concern regarding the preparation hypothesis is the suggestion that violent sexual behavior such as rape fulfills certain functions for men, namely exerting control over women and thereby increasing reproductive success, rather than being an instance of random behavior (Smith & Konik, 2011). I believe that sexual assault victims are not necessarily comforted by the idea that women have evolved an adaptive system in reaction to this male-dominant behavior. We might just as well explain to them that sexual responses depend on automatic processes that may unfold independently of values and preferences. I actually wonder how robust the finding is that women show a reflexive lubrication response during rape. The evidence so far has been based on clinical observations and responses to hypothetical scenarios rather than being established by a systematic line of research (Suschinsky & Lalumière, 2011). There is probably a large group of women who do not lubricate in a sexually violent situation. Yet, the idea of potential variation does not fit with evolutionary hypotheses that assume stability rather than individual differences. This is an important flaw of the preparation hypothesis. Averaging genital arousal scores across women and disregarding the impact of contextual influences and potential moderator variables does not acknowledge the variability and heterogeneity that characterizes sexual responding.

## Sexual Arousal as a Coordinated Pattern of Process Variables: The Need for Specific Predictions

One large benefit of the cognitive-emotional-motivational perspective on sexual arousal is that it generates specific predictions that can be empirically confirmed or falsified, and this is in contrast with evolutionary accounts that often lack scientific rigor and concreteness. Research has started to explore the role of automatic activation, attentional capture, maintained attention, attentional inhibition, memory, implicit and explicit appraisals, and approach-avoidance motivational tendencies in response to sexual stimuli (Bush, 2001; Dawson, Fretz, & Chivers, 2017; Dewitte, 2015, 2016; Janssen et al., 2000; Rodriguez-Nieto, Emmerling, Dewitte, Sack, & Schuhmann, 2019; Snowden & Gray, 2013; van Lankveld, Martin, Hubben, Creutz, & Verboon, 2013). These studies have informed us about the activation and regulation of the sexual system and how automatic genital arousal is involved in this.

The main issue with evolutionary explanations is that they do not deliver precise, detailed, and testable predictions (Confer et al., 2010; Hankin, 2013). The theory is inflated by post hoc explanations that spin into a theoretical and methodological loop, and therefore cannot be falsified. It sometimes feels as if the theory is used to consolidate difficult-to-explain findings into a “convenient” story. When certain observations are not in line with evolutionary predictions, additional hypotheses are proposed instead of questioning the theory in itself. For example, the finding that low cue-specificity is observed mainly in heterosexual and androphilic women, and that lesbian and gynephilic women produce less strong genital responses to male stimuli, is difficult to reconcile with the preparation hypothesis (Chivers, Seto, & Blanchard, 2007; Dawson et al., 2017). That evolutionary accounts cannot deal well with sexual orientation and same-sex behavior is a well-known flaw (Confer et al., 2010). Yet, to preserve the hypothesis, it has been suggested that a minimum level of vaginal lubrication is required to offer protection and that lesbian and gynephilic women still produce a sufficient-enough response. This really feels as if the hypothesis is being molded by adding a post hoc explanation that raises more questions than it offers solutions. What about this minimal requirement of vaginal lubrication and how does it fit with the dynamical nature of sexual system responding? Is this minimum level of vaginal lubrication specific to sexual stimuli? For example, when inserting a tampon or any other (sexual or non-sexual) object, it may be adaptive as well to start lubricating in order to prevent genital injuries.

In this context, it is worth noting that we actually lack a direct test of the preparation hypothesis because there is almost no evidence that relies on measures of lubrication. All conclusions are based on studies measuring vaginal blood flow, which is a different physiological process that likely has a different, not necessarily protective, function (Levin, 2003). As long as we do not understand the functional relation between vaginal vasocongestion and lubrication, no solid conclusions can be drawn on whether genital arousal primarily serves to prepare women for a (desired or undesired) sexual encounter.

## Sexual Arousal as a Multi-Dimensional Process: Practical Implications

Turning back to the idea that sexual responses are determined by different processes that frequently lack correspondence, it is difficult to draw specific inferences about sexual responding from any single level or determinant (Dewitte, 2016; Gross, 1998; Strack & Deutsch, 2004). In other words, studying the impact or function of one component requires studying its relationship with other components. At this point, the preparation hypothesis is limited in its explanatory value and scope because it focuses solely on the lubrication response without acknowledging any other related processes. The female genital response comprises of different physiological processes of which pelvic floor muscle function is at least as important to painless vaginal penetration as lubrication (Rosenbaum, 2007). Evidence has shown that pelvic floor muscle contraction is a reflexive and defensive response in the face of sexual danger (van der Velde, Laan, & Everaerd, 2001). How does the preparation hypothesis account for defensive pelvic floor responding and how does this reconcile with the need for a relaxed pelvic floor in order to allow (painless and pleasurable) penetration? This brings me to the case of female genital pain, which seems at odds with the evolutionary perspective, particularly because there is no solid proof that low genital arousal (at least as measured via vaginal vasocongestion) is involved, despite the experience of pain and the presence of vaginal/vestibular erythema (Brauer, Laan, & ter Kuile, 2006).

When elaborating on the practical and clinical value of evolutionary hypotheses, it becomes clear that this conceptual framework was primarily developed as a meta-theory that generates psychological explanations. Although some work has been done on clinical samples, the number of studies is limited and reliance on small sample sizes is common. In general, no significant differences have been found between women with and without sexual problems, supporting the idea that genital arousal serves to protect rather than fuel desire (Handy, Stanton, Pulverman, & Meston, 2018; Laan, van Driel, & van Lunsen, 2008; Suschinsky et al., 2019). One could question, however, the clinical value of this insight because it does not offer concrete targets of interventions. Or do we need to study the specifics of different sexual disorders to better evaluate the function and validity of the preparation hypothesis on female genital arousal? In my opinion, a cognitive-emotional-motivational perspective on sexual arousal is better suited to understand sexual problems. Given that sexual problems may be directed at specific phases in the cascade of sexual responding, understanding why and when people adopt specific regulatory strategies to deal with sexual arousal could help developing interventions that target defensive, inflexible, and/or negative types of sexual information processing (Dewitte, 2016).

In this respect, it is important to note that we lack a clear understanding of the adaptive or maladaptive value of concordant and discordant sexual responding and how this is implicated in sexual functioning (Suschinsky et al., 2019). Stronger feedback loops between the cognitive and physiological response systems may be preferable to increase sexual arousability and pleasure, but it may be less necessary in terms of enabling penetrative sex. It is thus plausible that the functional meaning of sexual concordance and how it relates to sexual and relational outcomes is different for men and women. To better understand the interrelation between genital and subjective sexual arousal as a function of gender or sexual function status, more research is needed on the underlying mechanisms of sexual concordance. In this context, the role of attention and the ability to become aware of one’s bodily signals of sexual arousal have been forwarded as potential explanatory mechanisms (Handy & Meston, 2018). If sexual arousal responding depends on interoceptive awareness of genital cues, then it may be more relevant to explore interoceptive and automatic attentional processes rather than studying the level or specificity of genital arousal responses. There is a large literature on interoceptive awareness that could help us answering many unresolved issues regarding the function and role of female genital arousal.

Another model that deserves more attention in research on sexual arousal is learning theory. Both appetitive and aversive conditioning mechanisms are likely involved in genital arousal responding, particularly when “prepared” sexual cues are presented (Brom, Both, Laan, Everaerd, & Spinhoven, 2014; Hoffmann, 2017). Furthermore, the activation of (genital) arousal in response to sexual stimuli would depend on automatic matching in memory, which assumes a possible role of learning processes (Hoffmann, 2017; Janssen et al., 2000). The fact that conditioning studies so far have not yielded robust effects does not mean that learning is not involved in sexual responding. A systematic line of research is needed to determine the exact parameters, stimulus modalities, and occasion setters of sexual conditioning, which will inform us about automatically activated genital (and subjective) arousal in response to various stimuli (Brom et al., 2014). It is also worth exploring whether genital and subjective sexual arousal are subject to different learning principles. Although evolutionary hypotheses are rather inflexible when it comes to acknowledging the role of environmental input, “learned” and “evolved” are not necessarily competing explanations (Confer et al., 2010). The learning framework offers concrete and testable predictions with direct implications for clinical interventions, thereby taking into account the role of differential learning experiences as a function of context and environmental input. Something that is learned can be relearned, and this holds promise for designing clinical interventions.

## Sexual Arousal as a Biopsychosocial Process

As a final comment on the preparation hypothesis, I want to address the lack of biopsychosocial integration regarding the role of female genital arousal. It is remarkable that no reference has been made to how biological markers such as hormones can trigger or prepare genital arousal and thereby facilitate or inhibit the protective function of vaginal lubrication (Bancroft, 2005; Khera, 2015). Knowing that testosterone increases sexual arousability (Bancroft, 2005; Khera, 2015), it may be relevant to broaden the scope of the preparation hypothesis and describe how the hormonal system has evolved to prepare the vaginal lumen for penetration. It is also remarkable that the social part of the biopsychosocial perspective on sexual responding has not been well addressed in evolutionary hypotheses. Stimuli that include contextual features such as partnered sexual interaction and relationship variables have been found to elicit a different pattern (i.e., higher level) of genital responding compared with solitary sexual acts or static and plain sexual stimuli (Chivers & Timmers, 2012; Dawson & Chivers, 2018). Furthermore, it has been demonstrated that the number of contextual cues increases the level of sexual concordance in women and that female genital arousal is higher in research environments that are closer to the natural context in which sex usually takes place (Bloemers et al., 2010; Chivers et al., 2010). This clearly points toward the moderating role of context, which is an observation that is difficult to reconcile with the preparation hypothesis.

This brings me to a fundamental weakness of current sex research. Although sexual arousal is most often experienced and expressed in a relationship, genital and subjective sexual responses are typically examined while participants are watching a sexual film clip on their own (Dewitte, 2014). Such a design more likely reflects solitary sexual responding and cannot capture the dynamic and dyadic nature of sexuality. Hence, if we want to create more valid models on sexual arousal, we need to include the partner in our measurement procedure. Embedding the sexual stimulus in a relational setting with the partner being present in the lab allows women to rely on partner-related cues for defining their sexual arousal state and to become more emotionally immersed in the sexual experience, resulting in stronger agreement between their subjective and genital arousal. This stronger agreement can take both positive and negative forms. That is, in case of positive sexual experiences with the partner in a supportive relationship climate, women may show higher genital and subjective sexual arousal. On the other hand, negative sexual experiences and relationship dissatisfaction or conflict may inhibit genital arousal responding and induce lower levels of subjective sexual arousal.

Considering the important role of context, we must consider that the sexual arousal responses observed in the lab are not an accurate reflection of real-life sexual responding in a relationship context. Although this does not necessarily mean that lab results are invalid, our conclusions on the manifestation, role, and function of genital arousal may be biased by procedural artifacts such as reactivity to the artificial lab context and the individual test situation. The preparation hypothesis does not fit well with a contextual approach that considers sexual arousal as a malleable, dynamic, and context-sensitive response. Another procedural concern is the stimulus material that has been used to elicit genital arousal, which is most often ego-dystonic rather than idiosyncratic and exogenous instead of endogenous. Allowing partners to sexually stimulate each other instead of passively watching a sexual movie may shed new light on the role of genital arousal and its association with subjective arousal and other components of sexual responding. Moreover, special attention needs to be paid to selecting control stimuli that show less systematic differences in terms of content, complexity, and salience. Using stimuli that gradually build up from neutral interactions between partners toward full-blown and explicit sexual interactions would advance our knowledge on the gradient of sexual arousal responding, thereby helping us understand the function and role of genital arousal.

## Conclusion

Although the Target Article provides arguments both in favor and against the preparation hypothesis, Lalumière et al. (2020) tend to be more supportive of an evolutionary explanation of genital arousal as a protective mechanism. In my opinion, the preparation hypothesis is not only unnecessary, it also faces some key challenges that limit its heuristic value and explanatory power and constrain its practical and clinical implications. The theory is not anchored in a solid line of research but rather serves as a post hoc explanation for a series of findings that are not entirely free from methodological concerns. I do want to emphasize that none of the ideas and theories I propose in this commentary are completely incompatible with an evolutionary explanation of female genital arousal. I am sure that Lalumière et al. would endorse these theoretical models as well. The same findings can be approached from very different angles that can easily coexist, but I do wonder whether the preparation hypothesis is sufficiently substantiated to actually advance our knowledge on the functional implications of female sexual arousal.
